# Determination of Selected Priority Pesticides in High Water Fruits and Vegetables by Modified QuEChERS and GC-ECD with GC-MS/MS Confirmation

**DOI:** 10.3390/molecules24030417

**Published:** 2019-01-24

**Authors:** Maciej Tankiewicz

**Affiliations:** Department of Environmental Toxicology, Faculty of Health Sciences with Subfaculty of Nursing and Institute of Maritime and Tropical Medicine, Medical University of Gdańsk, Dębowa Str. 23A, 80-204 Gdańsk, Poland; maciej.tankiewicz@gumed.edu.pl; Tel.: +48-58-349-1937

**Keywords:** pesticides, fruits and vegetables, QuEChERS method, gas chromatography

## Abstract

A modified quick, easy, cheap, efficient, rugged and safe (QuEChERS) method coupled to gas chromatography with electron capture detector (GC-ECD) was developed for simultaneous determination of selected electronegative pesticides in fruits and vegetables with high water content. The chosen compounds are commonly detected in fruit and vegetable crops, and some of their metabolites have even been found in human urine. In addition, some of them are known or suspected carcinogens according to the International Agency for Research of Cancer. Extraction and clean up parameters were optimized, thus the original QuEChERS method was modified to decrease solvent usage, in accordance with ‘green chemistry’ principles. The proposed methodology was validated in terms of selectivity, specificity, linearity, precision and accuracy. The obtained limits of detection (LODs) for all investigated pesticides ranged from 5.6 µg·kg^−1^ to 15 µg·kg^−1^ and limits of quantification (LOQs) from 17 µg·kg^−1^ to 45 µg·kg^−1^. The obtained data demonstrated the good reproducibility and stability of the procedure in the tested concentration range up to 10 mg·kg^−1^, with relative standard deviations (RSDs) lower than 10%. Recoveries for spiked pear samples at LOQ level for each pesticide were from 90% to 107% with RSDs lower than 9.6%. The suitability of the developed procedure was tested on various fruit and vegetable samples available on the market at different seasons. The proposed methodology is applicable for detection and monitoring of selected pesticides not only in fruits and vegetables with high water content, but also in samples containing large amounts of pigments and dyes.

## 1. Introduction

The use of pesticides provides unquestionable benefits in increasing agricultural production to grow the quantity and quality of food needed to sustain the human population. Food safety is one of the top priorities in public health protection and requires continuous development [1]. It is particularly important to ensure the safety of fresh food. This applies especially to fruits and vegetables as they are consumed directly and in the largest quantities without any processing. This is a source of exposure to harmful or toxic substances, such as pesticides. Despite the numerous advantages that pesticides have, they can also be hazardous and toxic substances, which pollute the environment and their fate and functioning remains unknown to a considerable extent [2,3]. Moreover, their residues are present in the treated products, which constitutes a potential risk for consumers [3,4,5].

Fruits and vegetables are susceptible to pests at any point in the production chain, from the field through storage and food consumption. Some pesticides are used before blooming, some while the fruit is growing and others after harvesting [6]. Post-harvest pesticides are the biggest source of synthetic pesticides in food [7]. Some hazards identified in fruit and vegetable production are due to incorrect application of pesticides on the part of the producer, in contradiction with good agricultural practice, and insufficient monitoring of their application. Contamination can also occur via run-off during floods or from contaminated soil or water [8,9].

To protect consumers from exposure to unacceptable levels of pesticide residues in food and feed, the European Commission has set maximum residue levels (MRLs), defined as the highest possible level of a pesticide residue that is legally authorized in food and feed [10]. Due to these low detection levels required to assess food safety and the complex nature of the matrices in which the target compounds are present, efficient sample preparation and trace-level detection and identification are important aspects of analytical methodologies [11,12].

The QuEChERS (Quick, Easy, Cheap, Effective, Rugged, Safe) method as an alternative to classical extraction techniques has proved its usefulness in food analysis. Initially, it was used for effective isolation of veterinary drugs in animal tissues. After realizing its great potential in the extraction of polar and particularly basic compounds, the original QuEChERS method was adapted in 2003 [13] for pesticide residue analysis in plant material, with great success. It has become the main analytical tool in most pesticide monitoring laboratories, because it can simultaneously achieve high quality results for a wide range of pesticides and it presents practical benefits desired by all laboratories over most traditional methods of analysis [14,15]. QuEChERS involves microscale extraction using acetonitrile and purifying the extract using dispersive solid-phase extraction (d-SPE). The consumption of sample and toxic solvents with the QuEChERS method is minimal. It requires fewer steps: no blending, filtration, large volume quantitative transfers, evaporation/condensation steps, or solvent exchanges required. This is very important, because every additional step complicates the procedure and may be source of systematic and random errors. By applying QuEChERS for the determination of pesticides in fruits and vegetables, matrix effects are eliminated and high recoveries of target analytes are possible [16]. The solvent of choice for the determination of pesticide residues in fruits and vegetables is acetonitrile [13,15,16], because the obtained extracts contain fewer interfering substances than the corresponding acetone and ethyl acetate extracts [17,18]. Additionally, acetonitrile can be separated fairly easily from water (salting out), therefore it is the preferred extraction solvent in the QuEChERS procedure [19,20,21]. The original method was modified in the following years to ensure efficient extraction of pH-dependent compounds (e.g., phenoxyalcanoic acids), in order to minimize the degradation of susceptible compounds (e.g., pesticides labile under alkaline and acidic conditions) and to expand the spectrum of matrices covered [22,23]. Of great importance was the introduction of buffering salts to improve recoveries of pH-dependant analytes. Buffering with citrate or acetate salts has been introduced in the first extraction/partitioning step to adjust the pH to a compromise value of 5 to 5.5, where most pesticides labile under acidic or alkaline conditions are sufficiently stabilized, allowing the analysis of various difficult commodities and pesticides [24]. In nonacidic matrices, such as lettuce, pesticides sensitive to a basic pH, like captan, folpet, dichlofluanid and chlorothalonil, were degraded. This problem was solved by addition of 0.1% acetic or formic acid solutions [25]. However, studies regarding pesticides that exhibit pH-dependent stability problems have not been reported so far, because each additional operation in the extraction process can cause loss of the analytes and thus lead to erroneous measurement results.

Pesticide analysis methodologies, usually in ultratraces range (µg·kg^−1^), require typically separative analytical techniques such as GC or HPLC, in one or two dimensions [14,15,16,17,18,19,20,21]. GC is the technique most widely used in simultaneous pesticide analysis because of its high-resolution capacity and the availability of selective detectors [26,27]. These detectors are applicable to classes of pesticides with similar properties. Thereby, very low limits of detection are obtained. The Electron Capture Detector (ECD) is very selective and sensitive to electronegative compounds, like organochlorine, organophosphate, and organonitrogen pesticides, some of these compounds in the parts per trillion (ppt) range [28]. However, there are only a few studies about application of the QuEChERS sample preparation method, modified or not, for pesticide analysis using gas chromatography coupled with ECD detector. This approach is a promising alternative to mass spectrometry (MS) due to its simpler operation, lower failure rates and lack of vacuum maintenance [29]. In addition, ECD detectors are widely available in both research and commercial laboratories.

The objective of the study was to develop an analytical procedure for routine analysis and simultaneous determination of selected electronegative pesticides in fruits and vegetables with high water content. For extraction, a modified QuEChERS sample preparation method was used and optimized. When selecting suitable solvents and sorbents used in the QuEChERS method, the content of water, pigments and sterols in tested fruits and vegetables were significant. As a screening test for presence of pesticide residues in tested fruit and vegetable samples, before the validation study, gas chromatography coupled with tandem mass spectrometry (GC-MS/MS) was applied. The selected pesticides are widely used in agriculture and their residues have been found in fruit and vegetable crops while some of their metabolites have been found in human urine [30]. Some of these pesticides are known or suspected carcinogens according to the International Agency for Research of Cancer [5,31]. Moreover, due to the origin of environmental samples from different countries, the selection of pesticides was based not only on the most frequently detected compounds in the country where measurements were conducted, but all over the world. The analytical procedure was validated, according with the requirements of the SANTE document, established by European Commission [32], and applied to monitor environmental fruit and vegetable samples, purchased at different seasons of the year from markets located in Gdańsk (Poland). For confirmation study, a second fused silica column was used. The developed methodology has proven to be sensitive and useful in determining pesticide residues in various fruits and vegetables, and provides a theoretical basis for analysis of other food samples in the future.

## 2. Results and Discussion

During the optimization of analytical procedure parameters, the first step was the selection of ECD detector operating conditions to allow efficient pesticide determination. Subsequently, the terms of the separation process (type of chromatography column, the composition and flow rate of carrier gas, temperature programme) were selected, and in the final stage the extraction conditions were chosen. Lastly, the developed analytical procedure was validated and applied to analysis of environmental samples.

**Figure 1:**
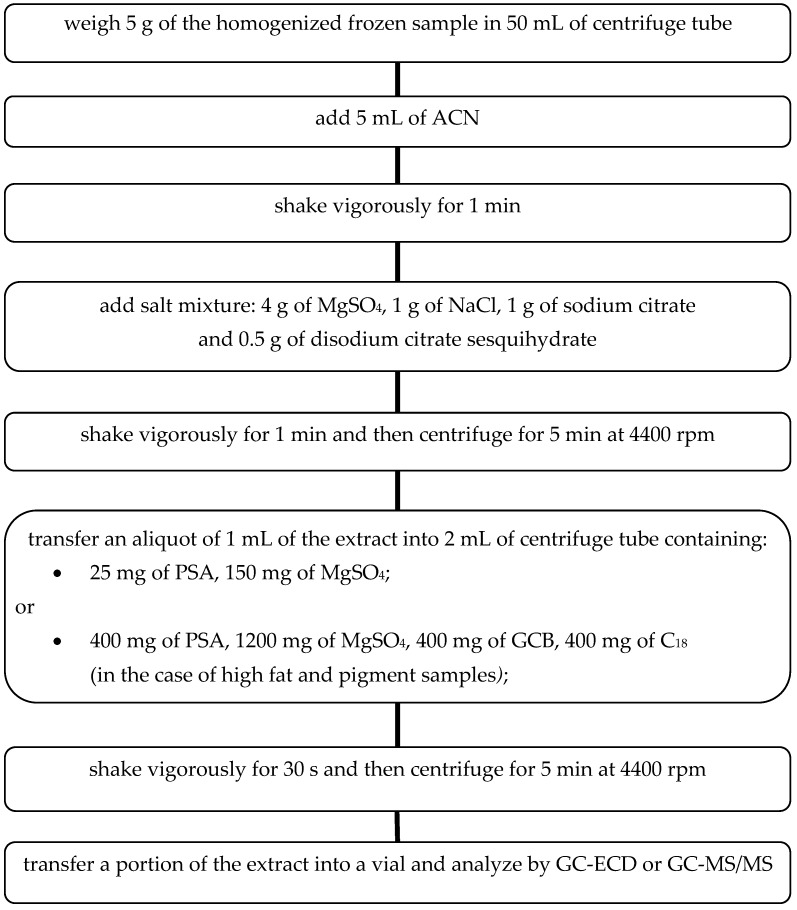
Optimized QuEChERS procedure.

Different parameters affecting the QuEChERS sample preparation method were evaluated to optimize the procedure based on the European Method prEN 15,662:2008 [19] for the determination of pesticide residues. The extraction and cleanup steps of the QuEChERS method are summarized in the Figure 1.

The experimentally selected conditions for the sample preparation process ensure efficient and effective extraction of pesticides from selected fruits and vegetables. Moreover, it can be successfully applied to other groups of fresh food. In the case of high fat and pigmented samples, addition of graphitized carbon black (GCB) and C_18_-reversed phase silica gel at the extract purification step is recommended. Large pigment molecules may clog the chromatographic column and interfere with the separation process. At the same time, in the case of planar pesticides determination, the use of GCB should be avoided. These can be retained by carbon, thereby decreasing the recovery. GCB removes pigments, sterols and non-polar interferences, and may be added to assist the cleanup of intensively colored extracts due to a high content of carotenoids (e.g., carrots, red sweet pepper) or chlorophyll (e.g., spinach, rucola, lettuce). C_18_ or C_8_ reversed-phase silica sorbents (400 mg per 1 mL of extract is recommended) may be used to adsorb lipids and non-polar interferences in extracts with remaining fats. Co-extracted waxes and fats can be effectively separated from the extract by cooling in the freezer (>1 h) and then centrifugation.

### 2.1. Optimization of the Sample Preparation Method

Commercially available pre-packaged QuEChERS kits (Agilent Technologies, Santa Clara, CA, USA) were chosen to develop the extraction and clean-up steps. Apple samples were selected to carry out the optimization. For the extraction step, buffered kits are recommended as a first choice, even if weakly acid/basic sensitive pesticides are included in the target analytes group. Two classes of buffered kits are offered by different companies, which are applicable to the European prEN 15662:2008 [19] and American AOAC 2007.01 [33] official methods guidelines. The European buffered method was selected because it is more commonly used.

There is a large variety of selective dispersive SPE (d-SPE) kits (Agilent Technologies, Santa Clara, CA, USA) for cleanup purposes, including different amounts of PSA, MgSO_4_, GCB and C_18_. Each one of them is recommended for the cleaning up of the extracts of different groups of fruits and vegetables containing different co-extracted compounds. The most commonly used kits for cleanup step are presented in the Supplementary Materials (Table S1).

#### 2.1.1. Effect of Clean-up and d-SPE Composition

Due to the recommendation to use acetonitrile (ACN) as the extractant in a ratio of 1:1 to the sample mass [13,19,25,33], the same proportion was also applied in this study. This ensures the effective isolation of pesticides and minimizes the process of passing interfering substances present in the matrix to the organic layer. Therefore, 10 g of apple sample spiked with pesticides at the concentration level of 2 mg kg^−1^ was extracted with 10 mL of ACN. The tube was shaken by hand for 1 min and then a mix of salts containing 4 g MgSO_4_, 1 g NaCl, 1 g sodium citrate, 0.5 g disodium citrate sesquihydrate, was added and the tube was shaken again for 1 min, and centrifuged at 4400 rpm for 5 min.

To evaluate the effect of the d-SPE composition, two different selective dispersive SPE kits dedicated for general fruits and vegetables, and for fruits and vegetables with fats and waxes were selected (Agilent Technologies, Santa Clara, CA, USA). Among all possible kit choices, the softest and harshest mixtures were chosen: (1) 1 mL of extract cleaned up in 2 mL cartridges containing 25 mg PSA, 150 mg MgSO_4_, (2) 8 mL of extract cleaned up in 15 mL cartridges containing 400 mg PSA, 1200 mg MgSO_4_, 400 mg GCB, 400 mg C_18_. Cartridges were shaken by hand for 30 s and after that centrifuged at 4400 rpm for 5 min. The detector signals of both cleaned up extracts and untreated extracts were compared.

When 15 mL of clean-up kit was used, only one peak corresponding to α-endosulfan was detected. In addition, the detector response was lower than in the case of a mixture of amine and anhydrous magnesium sulfate. The other peaks corresponding to pesticides and the matrix interferences disappeared. This can be explained by the fact that the composition of the 15 mL clean-up d-SPE kit is intended for fruits and vegetables with high pigment and fat content, and since these components are not present in the matrix, the GCB and C_18_ remove not only the matrix co-extracts, but also the pesticides in the extract.

**Figure 2:**
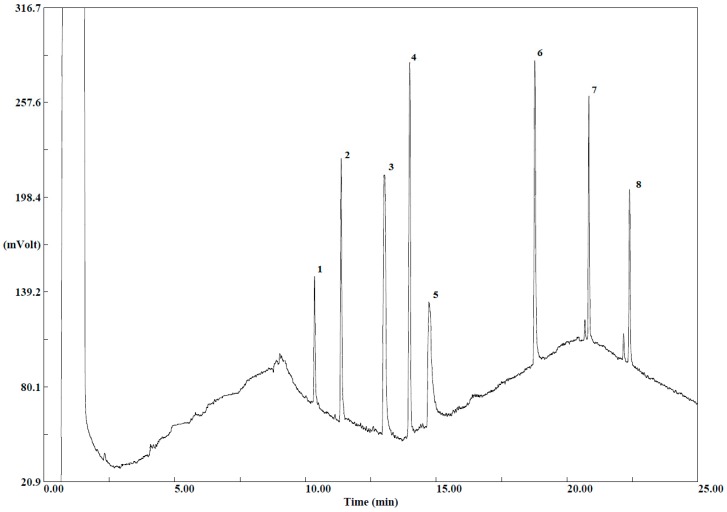
Chromatogram obtained for 10 g of spiked apple sample at the concentration level 2 mg·kg^−1^ for each pesticide by using QuEChERS-GC-ECD, **1:** Tolclofos-methyl, **2:** Chlorpyrifos, **3:** Captan, **4:** α-endosulfan, **5:** Imazalil, **6:** Phosalone, **7:** α-cypermethrin, **8:** Deltamethrin.

When comparing the chromatograms for untreated extracts and those treated with a 2 mL clean-up kit, it has been observed that interferences originating from the matrix at the beginning of the chromatogram for the 2 mL clean-up were less disturbing, and the shape of peaks was improved. Moreover, the final extracts were clear and color intensity was less in comparison with the extracts before the cleanup step. Purification with d-SPE ensures fewer artefact peaks and hence, improving chromatography. In addition, interfering compounds that are removed during this step can also affect the lifetime of the chromatography column and the detector. Thus, the 2 mL d-SPE kit for general fruits and vegetables containing 25 mg PSA, 150 mg MgSO_4_ was chosen for the cleanup of extracts in this study. The obtained chromatogram is presented in the Figure 2. Therefore, the choice of d-SPE composition must take into account the kind of fruit and vegetable, because choosing more aggressive phases can lead to the loss of target compounds.

#### 2.1.2. Optimization of Sample Amount

Two different amounts of sample were assayed, 10 g and 5 g, to study the differences in the extraction of matrix components. Extracts were obtained following the same procedure and reagents composition.

The presence of matrix interferences in the final extract leads to a deviation of the baseline of the chromatogram from the horizontal line. Between 5 minutes and 10 minutes of retention time (Figure 2) a cyclical baseline was observed. At that time, none of the analytes eluted from the column. When comparing both chromatograms, the baseline for the 5 g sample was less disturbed. Additionally, the presence of matrix interferences in the final extract of the 5 g sample was lower than for the 10 g sample. Nevertheless, both chromatograms presented similar profiles and therefore it can be concluded that the extraction of pesticides is not influenced by the amount of sample. The matrix influence was also examined by comparing chromatograms obtained for standard mixture of pesticides and spiked apple sample at 1.0 mg·kg^−1^. The cyclical baseline and artefact peaks were not observed in the chromatogram for standard mixture. Moreover, a conducted sample dilution test helped to reduce the matrix effect. On this basis, it was stated that the increase of baseline on the chromatograms was caused by the matrix effect.

The use of 5 g of sample reduces the amount of ACN used for the extraction by 50%, since only 5 mL of solvent is needed compared to the 10 mL of the original QuEChERS method for the 10 g sample. This decrease in solvent use is consistent with the principles of ‘green chemistry’ as well as providing a lower baseline in detector response; therefore, the use of 5 g of samples was chosen for this study.

#### 2.1.3. Effect of Sample Dilution

**Figure 3:**
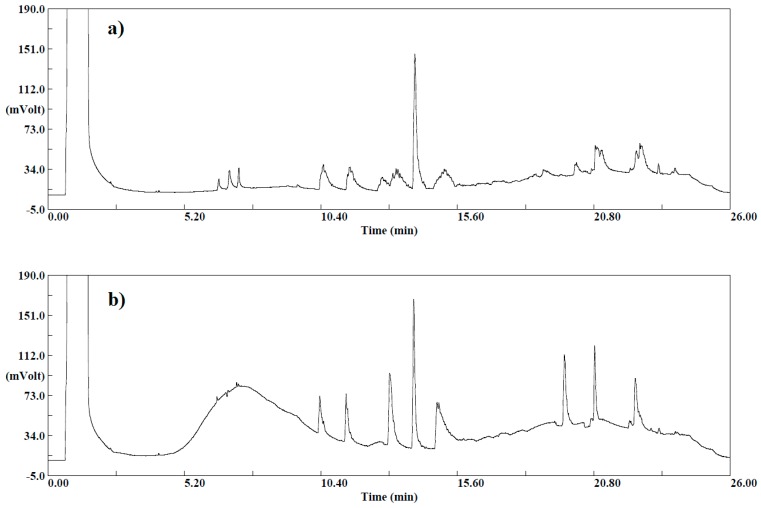
Chromatograms obtained for the extracts from: (**a**) 5 mL (1:10) of sample solution and (**b**) 5 g of the original sample by using QuEChERS-GC-ECD.

One gram of sample (apple) was diluted with 9 mL of deionized water, producing a 1:10 solution. Subsequently, 5 mL of this solution and 5 g of the original sample were spiked with standards and extracted with 5 mL of ACN, following the same procedure. The obtained chromatograms for both extracts are presented in the Figure 3.

Sharper peaks and higher detector responses were observed in the chromatogram corresponding to 5 g of the original sample extract compared with the 5 mL diluted sample, while the interference of the matrix co-extracts (expressed as lack of cyclical baseline) is lower in the chromatogram of the diluted 5 mL sample. This enhancement of the signal of analytes is an effect produced by the matrix and is commonly observed in GC. In this study, the extraction was performed from 5 g of the original sample to benefit from the signal improvement due to the matrix effect, and the effect of dilution was considered in case the dilution of any real sample would be needed to keep the concentration in the calibration range. Moreover, it should be noticed that fruits and vegetables with low water content (<80%) require the addition of water before extraction to achieve a total of 10 g of water (when 10 g of sample is employed). In products with a water content <25% (e.g., dried fruit), the sample amount may have to be reduced (e.g., 1–5 g) and water can be added before processing to assist comminution.

#### 2.1.4. Evaluation of the pH Adjustment Effect

Since pesticides are a numerous and diverse group of chemical compounds, the adjustment of the pH to 5.0–5.5 values, after extraction and clean-up steps, increases the stability of some sensitive pesticides, while it can adversely affect others with different chemical characteristics. After treatment with PSA, the pH of the extract increases above 8, compromising the stability of base sensitive pesticides. Simultaneously, compounds with acidic groups may interact with the amino-sorbent. Thus, if such pesticides are scrutinized, their analysis should be performed directly from the raw extract after centrifugation and prior to the clean-up step.

In this study, the effect of pH adjustment was evaluated by comparison of detector signals on the chromatograms. During the experiments the pH value was adjusted to 5.0–5.5 firstly after the extraction with ACN by addition of NaOH solution and after the clean-up step by addition of acetic acid solution. The detector response of pH adjusted extract was found to be lower than for non-pH adjusted extract. This could be explained by the difficulty of pH adjustment by adding drops of base/acid solution to the small extraction volume (about 1 mL), producing dilution of the final extract and probably pesticide losses. Therefore, the pH values of extracts were not adjusted for further study.

On the other hand, the main advantage of pH adjustment is increasing stability of the extracts for several days, which was unnecessary in this study because extractions and injections were programmed to be performed on the same day. If the injection of extracts stored for more than one week would be needed, the pH adjustment should be taken into consideration, because captan could present stability problems.

### 2.2. Method Validation

In order to validate the analytical methodology, the GC-ECD was firstly calibrated. For this purpose, standard mixtures spiked at various concentration levels (described in Section 3.1) were injected three times directly into the GC column. Subsequently, the calibration curves were plotted separately for each analyte and the limits of detection (LODs) and quantification (LOQs) were calculated. The next step was to determine the initial values of pesticide recoveries after the QuEChERS step. For this purpose, spiked apple samples at the 1.0 mg·kg^−1^ level of pesticides were analyzed five times. Next, the recovery values of analytes were calculated, which ranged between 70–120% and relative standard deviation values were lower than 20%. The obtained results were satisfactory and met all the requirements set for analytical procedures; therefore, the whole methodology was validated. For this purpose, apple samples spiked at three concentration levels were prepared: at the LOQ level specified in the first stage, 10 times of the LOQ level (these values were different for each analyte) and at 10 mg·kg^−1^ for all compounds. Such high concentration levels were dictated by MRL values [10]. For example, MRL for captan in pome fruits is 10 mg·kg^−1^. That was one of the reasons to develop a new methodology, because the existing analytical methods are unavailable at high concentration levels. Samples prepared in this way were analyzed five times and the basic validation parameters were calculated, as shown in Table 1.

To verify the validity of the developed procedure, recovery studies were performed. The extractions were carried out from samples spiked at two concentration levels: corresponding to the LOQs for each pesticide and multiples (10 times) of LOQs. Higher concentrations were dictated by the values of the highest allowable concentrations for individual compounds depending on the respective matrix. In order to check the effect of different matrices on the analysis, the sample type was changed. At this stage, pears were selected for testing. Properly spiked pear samples were analyzed seven times. Based on the obtained results (Table 2), recovery values and relative standard deviation (RSD) values were calculated, which are the measure of the repeatability of the methodology. Recoveries for spiked pear samples at LOQ level for each pesticide were from 90% to 107% with RSDs lower than 9.6%. For higher concentration levels that were 10 times greater than LOQ values, the recovery was in the range of 89–107% with RSDs between 3.2% and 5.7%. The average recovery for both spike levels of pesticides proved to be included in the range 70–120%, with the RSD ≤ 20%, in accordance with the SANTE validation requirements [32].

### 2.3. Environmental Samples

In order to check the suitability of the developed procedure, various fruit and vegetable samples available on the market at different seasons were analyzed. The course of analytical procedure was the same as during the method validation step. Each sample type was analyzed five times. The signals obtained in the chromatograms were integrated, thus the peak areas were received. Then, the concentrations of detected pesticides were calculated using the equations of calibration curves according to the following formula: peak area = b × concentration + a → concentration = (peak area − a)/b. For each sample type, the mean concentration values and RSDs were calculated. The obtained results for samples were compared with the highest maximum residue limits specified in legal regulations [10]. Only four pesticides were detected in environmental samples. Table 3 presents the values of pesticide concentrations together with the uncertainty of measurement expressed as RSD values obtained for environmental samples using QuEChERS-GC-ECD. Due to the differences in analyte recovery values, a student′s *t*-test has been applied. One-sample *t*-test to estimate the confidence interval for the mean value was used. Subsequently, it was checked whether the calculated concentration value in the environmental sample is within the expected value range. Based on the student′s *t*-test results, the individual results obtained for environmental samples did not show statistically significant differences [34]. 95% of the results were in the range of expected values.

Based on the data presented in Table 3 it can be observed that the highest acceptable value of tolclofos-methyl in cucumbers and mushrooms was exceeded. Detected concentrations of phosalone in white radish, red pepper and tomatoes were higher than those established in legal regulations. In addition, the residue of deltamethrin in broccoli was also higher than the permissible value. An exemplary chromatogram obtained for red pepper sample contaminated by phosalone is presented in the Supplementary Materials (Figure S1). It should be emphasized that the analyzed vegetables and fruits were not washed before the analysis, which may have a significant impact on the risk of harm to the consumer. Furthermore, some of them are consumed directly without any processing (for example: peeling, scraping, boiling or heating), which may be an additional risk of exposure. Most pesticides are efficiently removed from fruit and vegetable samples by washing with tap water [35]. Moreover, samples were tested during the non-vegetative period and most of them were imported, thus there was a greater probability of detecting the presence of pesticides [7,8].

## 3. Materials and Methods

### 3.1. Chemicals and Reagents

Pesticide standards (α-cypermethrin, α-endosulfan, captan, chlorpyrifos, deltamethrin, imazalil, phosalone and tolclofos-methyl), each at 100 mg·L^−1^ in methanol (MeOH) or acetonitrile (ACN), were purchased from ULTRA Scientific (North Kingstown, RI, USA).

LC-grade acetonitrile and methanol were obtained from Merck (LiChrosolv^®^, Darmstadt, Germany). Acetic acid, purity > 99.5%, from POCH S.A. (Gliwice, Poland); sodium hydroxide from STANLAB (Lublin, Poland); ultrapure water Milli-Q gradient A10 from Millipore Corporation (Molsheim, France) were used in this study. Pre-weighted kits for extraction, containing anhydrous magnesium sulfate (MgSO_4_), sodium chloride (NaCl), sodium citrate (C_6_H_5_Na_3_O_7_), and disodium citrate sesquihydrate, and pre-weighted clean-up mixtures in polypropylene tubes containing different amounts of MgSO_4_, primary secondary amine (PSA), graphitized carbon black (GCB), and silica reversed-phase sorbent C_18_ were purchased from Agilent Technologies (Santa Clara, CA, USA).

Individual working standard solutions at 1 mg·L^−1^ and 10 mg·L^−1^ were prepared in ACN by dilution of the corresponding stock standard solutions. They were used for spiking of samples in recovery studies and calibration purposes. Multicompound standard solutions of selected pesticides at 10 mg·L^−1^, 2 mg·L^−1^, 1 mg·L^−1^, 0.1 mg·L^−1^, 0.05 mg·L^−1^, 0.025 mg·L^−1^, and 0.01 mg·L^−1^ concentration levels for each compound were prepared in ACN for optimization and precalibration purposes. Two multicompound standard solutions were prepared in apple matrix blank extract at the LOQ concentration level obtained from the precalibration of the GC-ECD, and the other solution at a concentration ranging from 2–10 times of LOQ level for each compound. These solutions were used for calibration purposes according with SANTE procedure [32]. Multicompound standard solution at LOQ level was also prepared in pure ACN for method validation purposes. All solutions were stored in the dark at −18 °C.

### 3.2. Fruit and Vegetable Samples

Samples were collected from locations where they were sold for consumption. Apple, pear, nectarine, tomato, cucumber, mushroom (champignon), red beet, carrot, potato, white radish, red pepper and broccoli samples were purchased from local fresh markets located in the city of Gdańsk (Poland), and transported to the laboratory at 4 °C. Fresh vegetables and fruits came from both local farmers and producers as well as from imports. The study was started during the vegetative season, when the plants fruited and matured in a natural way. More batches of products were bought at different frequencies during the autumn and winter seasons.

Laboratory samples were taken in accordance with Directive 2002/63/EC [36]. Each unit of the laboratory sample was chosen from a random position in an accessible part of the lot and the minimum number of units was determined according with the size and weight of the commodity, as shown in Table 4.

Preparation of samples and sub-samples was performed in the shortest practicable time before any visible deterioration occurred. Fruit and vegetable samples (1–2 kg of each commodity), without cleaning, were cut into pieces and then homogenized with appropriate chopper and blender devices. For validation studies, the purchased fruit and vegetable samples were first analyzed for presence of pesticide residues by using GC-MS/MS. Subsequently, portions of non-pesticide-containing fruits and vegetables were further tested. Thus, adulteration was avoided. Analytical samples were weighted in 50 mL centrifuge tubes and stored in the freezer until analysis. Apple samples were chosen as representative matrix for method validation, and pears for the development of recovery studies. The characteristics of the examined fruits and vegetables are presented in Table S2 in the Supplementary Materials.

### 3.3. QuEChERS Procedure

Extractions were performed from frozen samples to minimize possible sensitive pesticide losses due to the heat produced in the sample after salt addition. The 5.0 ± 0.001 mL of ACN was added to the centrifuge tube and then shaken for 1 min. After that, the mixture of salts containing 4 g MgSO_4_, 1 g NaCl, 1 g sodium citrate and 0.5 g disodium citrate sesquihydrate, was poured and immediately shaken for 1 min, and then centrifuged at 4400 rpm for 5 min. An aliquot of 1.0 ± 0.001 mL of the upper layer (organic phase) was transferred to a 2 mL polypropylene centrifuge tube prefilled with 150 mg of MgSO_4_ and 25 mg of PSA. Subsequently, it was mixed for 30 s and after that centrifuged at 4400 rpm for 5 min. The clean extract was next transferred to a 1.5 mL glass vial and analyzed by GC-ECD or GC-MS/MS. Samples were normally analyzed by the same day and kept in the fridge when analysis was not running.

### 3.4. Chromatographic Analysis

As a screening test for presence of pesticide residues in tested fruit and vegetable samples, before the validation study, GC-MS/MS was applied. Thus, false positives and additional errors were eliminated. GC-MS TQ 8040 (Shimadzu Corp., Kyoto, Japan) equipped with split/splitless injector operating in a splitless mode at 250 °C and Zebron^TM^ ZB-Multiresidue-1 fused silica column (30 m × 0.25 mm × 0.25 µm film thickness; phase specially designed for the separation of all types of pesticides) supplied by Phenomenex (Torrance, CA, USA) connected to “LabSolutions” software (version 4.45, Shimadzu Corp., Kyoto, Japan) extended with Pesticide Smart Database (version 1.03, Shimadzu Corp., Kyoto, Japan) was used. The injection volume of 2 µL was selected for all analyses. The carrier gas was helium (purity 99.9999%) maintained at a constant flow of 1.0 mL min^−1^. Chromatographic separation in standard conditions was performed, with a temperature program ranging from 70 °C to 280 °C (at 12 °C min^−1^) and held at 280 °C for 4 min. The following MS conditions were used: ion source temp. 200 °C, interface temp. 280 °C, ionization voltage 70 V, emission current 150 µA and solvent cut time 4 min.

A HP-6890 series GC gas chromatograph (Hewlett-Packard, Palo Alto, CA, USA) equipped with on column injector coupled with an electron-capture detector (ECD-63Ni, Finnigan, Waltham, MA, USA) was used. The injector temperature was the same as the initial GC oven temperature and set at 70 °C. The chromatographic column Zebron ZB-5 (30 m × 0.25 mm × 0.25 µm of 5% phenyl- 95% dimethylpolysiloxane) supplied by Phenomenex (Torrance, CA, USA) was used. Hydrogen (CP grade, 99.9999%) from a gas generator HG2600 (Claind, Tremezzo, Italy) was used as carrier gas. The carrier gas flow was maintained at 1 mL·min^−1^, with constant flow conditions being observed throughout. Nitrogen (99.999% purity) from Messer (Chorzów, Poland) was used as a make-up gas at a flow rate of 40 mL min^−1^. The temperature program was as follows: 70 °C, 15 °C min^−1^ to 200 °C (hold 5 min), then 15 °C min^−1^ to 300 °C (hold 4 min). The detector temperature was 300 °C. The 2 µL of sample was injected into the GC system by using a 5 µL syringe (Hamilton, Reno, NV, USA).

For confirmation study of the developed analytical methodology, a second fused silica column was used. The certainty of identification based only on retention times is often not sufficient. As an independent confirmatory method, the separation process conditions were changed using a GC column with a different stationary phase [37]. In this study, a ZB-XLB capillary column (30 m × 0.25 mm × 0.25 µm film thickness) supplied by Phenomenex (Torrance, CA, USA) was used. This column provides alternative selectivity for the phase used initially and is recommended by the manufacturer for confirmation in pesticide analyses.

### 3.5. Method Validation and Statistical Calculations

Validation of the method is essential in order to assess that the developed methodology meets the legal requirements. For this purpose, the experimental sequence proposed in the SANTE official technical guideline for pesticide residues control in food and feed in the European Union (EU) was performed [32]. Apple samples were chosen as a representative matrix for method validation and pear samples for the development of recovery studies. The change of matrices ensures that false positives or false negatives are not reported and their components (e.g., pigments, carbohydrates, acids, starch, sugar, essential oils, fat, etc.) do not interfere in the separation process. For validation studies, reagent blank, unspiked sample and 5 replicates of spiked samples at LOQ, 2–10 times of LOQ levels (from pre-calibration study) and at 10 mg·kg^−1^ for each pesticide where prepared from homogenized apple sample, according with the optimized QuEChERS procedure. The LOQ and 2–10 times of LOQ levels for the methodology were calculated for each pesticide from the LOQ pre-calibration values of the GC-ECD equipment. Validation parameters were determined and verified against the criteria obtained from obtained sequence data.

In order to compare the significance of differences between the expected value of concentration and the determined in samples, the Student’s *t*-test (ƒ = n − 1, α = 0.05, t_crit._ = 2.7764) has been applied. This test enables to examine whether individual measurement results are within the expected value range [35]. This test was used at the stage of environmental samples determination.

LODs and LOQs were calculated based on the residual standard deviation of the calibration function (S_a_) and the slope of the calibration curve (b) according to the formulas LOD = 3.3 × (S_a_/b) and LOQ = 10 × (S_a_/b), respectively. In order to estimate the method suitability, the intra- and inter-day precisions of injection were evaluated by examining the retention times and peak areas of analytes [38]. Standard mixture solutions at three different concentration levels (low, medium and high) were injected (with five replicates) twice per day and on two different days. The obtained results indicated that RSD values for retention time and peak area were less than 7%. Repeatability was described by the coefficient of variability (CV%) [39,40].

## 4. Conclusion

In this paper, a new analytical procedure for routine analysis and simultaneous determination of selected electronegative pesticides was developed. For extraction, a modified QuEChERS sample preparation method was used. Extraction and clean-up parameters were optimized, and the original QuEChERS method was modified to decrease solvent use, in accordance with the principles of ‘green chemistry’. The analytical procedure was validated, according with the requirements of the SANTE document, and applied to monitor environmental fruit and vegetable samples with high water content.

The obtained validation data has confirmed that QuEChERS extraction ensures satisfactory results for all investigated pesticides. However, it should be noted that the selection of an appropriate technique for isolation and/or enrichment of analytes depends on the group of analyzed pesticides, their physicochemical properties and content in tested sample. In addition, the matrix composition and presence of interfering substances may significantly impede effective isolation and interfere with the detector. Based on the results obtained from environmental sample analysis, it has been proven that the proposed procedure is suitable not only for fruits and vegetables with high water content, but also for samples containing large amounts of pigments and dyes. In future works the extension of this method to other fruits and vegetables matrices and pesticides is feasible.

## Figures and Tables

**Table 1:** Basic validation data for selected pesticides obtained by using QuEChERS coupled with GC-ECD.

Analyte	Retention Time [min]	Equation	Coefficient of Determination R^2^	Limit of Detection LOD [mg·kg^−1^]	Limit of Quantification LOQ [mg·kg^−1^]	Relative Standard Deviation RSD (*n* = 5) [%]	Linearity Range [mg·kg^−1^]	Coefficient of Variability CV [%]
Tolclofos-methyl	10.33	y = 6697360.2x − 36613.5	0.9973	0.0081	0.024	8.8	0.024–10	2.2
Chlorpyrifos	11.35	y = 12727894x + 146752.5	0.9960	0.012	0.036	10	0.036–10	3.6
Captan	12.99	y = 16130151.2x − 1126031.1	0.9953	0.0073	0.022	7.6	0.022–10	7.6
α-Endosulfan	13.93	y = 20287013.6x − 22439.5	0.9996	0.0056	0.017	4.6	0.017–10	1.1
Imazalil	14.83	y = 17097689.2x + 303198.2	0.9867	0.0096	0.029	9.7	0.029–10	7.6
Phosalone	18.76	y = 11311475.2x − 174828	0.9979	0.015	0.045	5.6	0.045–10	2.7
α-Cypermethrin	20.82	y = 9491107.2x + 1170600.3	0.9972	0.0091	0.027	7.6	0.027–10	6.2
Deltamethrin	22.36	y = 7825560.6x − 64754.4	0.9988	0.0076	0.023	5.5	0.023–10	2.2

**Table 2:** Recovery mean values for each pesticide at LOQ and 10 × LOQ spiking levels.

Analyte	Concentration Levels of Spiked Samples [mg·kg^−1^]		Recovery [%]		RSD (*n* = 7) [%]	
	LOQ	10 × LOQ	LOQ	10 × LOQ	LOQ	10 × LOQ
Tolclofos-methyl	0.030	0.50	107	95	6.5	3.9
Chlorpyrifos	0.040	0.50	90	89	8.7	4.2
Captan	0.030	0.50	109	95	5.5	5.7
α-Endosulfan	0.020	0.50	100	104	8.2	4.2
Imazalil	0.030	1.0	104	97	5.9	3.2
Phosalone	0.050	0.50	102	91	2.6	4.0
α-Cypermethrin	0.030	0.50	104	105	9.6	3.3
Deltamethrin	0.030	0.50	98	107	5.6	4.1

**Table 3:** Mean values of pesticide concentrations with uncertainty determined in environmental samples and MRLs [10].

Concentration ± RSD (*n* = 5) [mg·kg^−1^]		Environmental Samples			
		Tolclofos-methyl	Imazalil	Phosalone	Deltamethrin
commodity	White radish	-	-	**0.27 ± 0.01**	-
	Broccoli	0.079 ± 0.013	-	<LOQ	**0.29 ± 0.02**
	Cucumber	**0.12 ± 0.04**	0.094 ± 0.0023	0.046 ± 0.0022	-
	Red pepper	0.070 ± 0.0094	-	**0.23 ± 0.01**	-
	Nectarine	-	<LOQ	<LOQ	0.026 ± 0.0014
	Mushroom	**0.080 ± 0.007**	-	-	-
	Tomato	-	-	**0.17 ± 0.01**	-
	Carrot	0.10 ± 0.0065	<LOQ	<LOQ	-
	Potato	0.064 ± 0.011	-	-	-
	Red beet	-	-	<LOQ	-
maximum residue levels [mg·kg^−1^]					
commodity	White radish	0.1	0.05	0.05	0.05
	Broccoli	0.5	0.05	0.05	0.1
	Cucumber	0.05	0.2	0.05	0.2
	Red pepper	1.0	0.05	0.05	0.2
	Nectarine	0.05	0.05	2.0	0.15
	Mushroom	0.05	0.05	0.05	0.05
	Tomato	1.0	0.5	0.05	0.3
	Carrot	0.5	0.05	0.05	0.05
	Potato	0.2	3.0	0.05	0.2
	Red beet	0.5	0.05	0.05	0.05

“-” means no signal detected; bold numbers indicate concentrations over the MRLs and their respective limits.

**Table 4:** Description of primary samples and minimum size of laboratory samples.

Commodity Classification	Samples	Nature of Primary Sample to be Taken	Minimum Size of Each Laboratory Sample
medium sized fresh products, units generally from 25 to 250 g	apple, pear, nectarine, tomato, cucumber, mushroom, red beet, carrot, potato, white radish	whole units	1 kg (at least 10 units)
large sized fresh products, units generally >250 g	red pepper, broccoli	whole units	2 kg (at least 5 units)

## Supplementary Materials

The following are available online, Figure S1: Chromatogram obtained for red pepper sample by using QuEChERS-GC-ECD, 1: Phosalone, Table S1: Commercially available kits for clean-up step, Table S2: Fruits and vegetables chosen for the study according to the SANTE guideline.
