# Supplementary material for: Determination of Selected Priority Pesticides in High Water Fruits and Vegetables by Modified QuEChERS and GC-ECD with GC-MS/MS Confirmation
Source: Molecules. 2019 Jan 24;24(3):417. doi: 10.3390/molecules24030417 (PMC6384567; doi:10.3390/molecules24030417)
Supplement: Supplementary file 1 [file molecules-24-00417-s001.pdf]

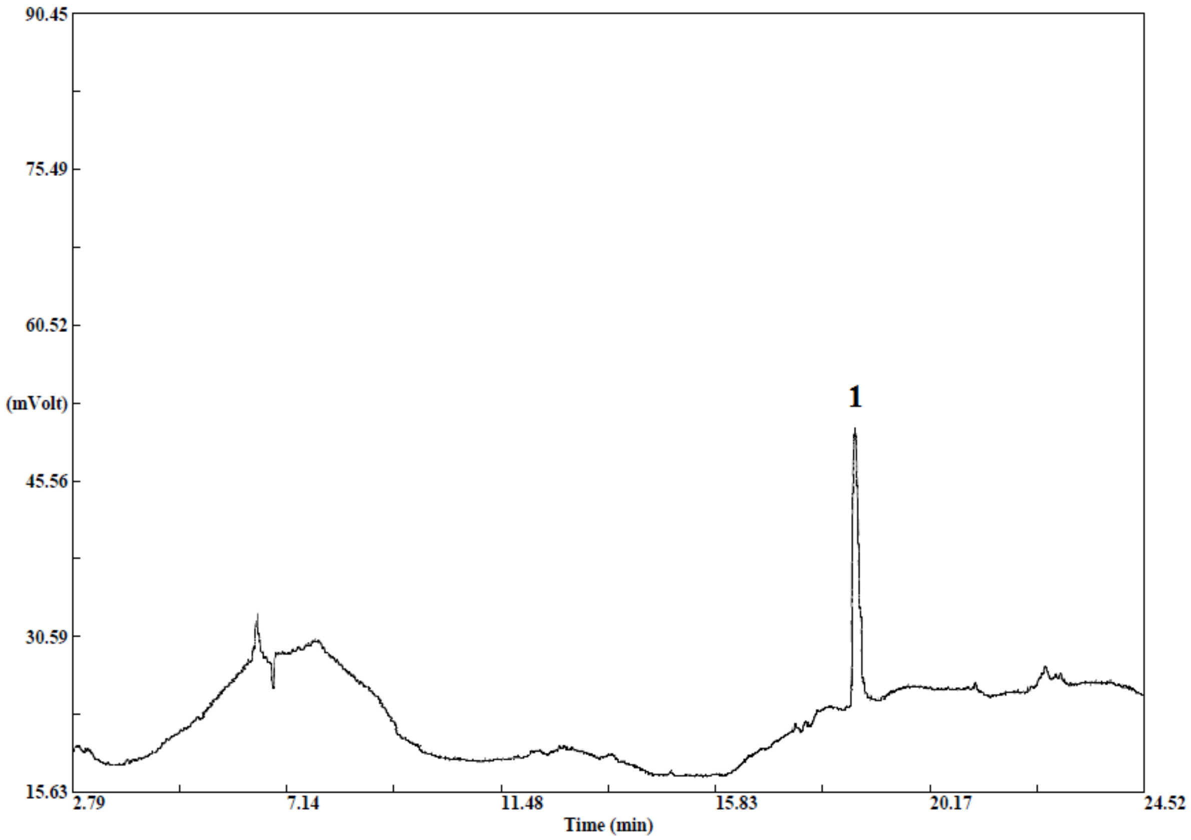

Figure S1. Chromatogram obtained for red pepper sample by using QuEChERS – GC-ECD, **1:** Phosalone.

Table S1. Commercially available kits for clean-up step [1-3]

Kit	Tube size [mL]	Volume of extract needed [mL]	dSPE sorbent composition [mg]	Applicable to official method
**General fruits and vegetables**	2	1	50 PSA, 150 $MgSO_4$	AOAC 2007.01
	15	8	400 PSA, 1200 $MgSO_4$	
	2	1	25 PSA, 150 $MgSO_4$	prEN 15662
	15	6	150 PSA, 900 $MgSO_4$	
**Fruits and vegetables with fats and waxes**	2	1	50 PSA, 150 $MgSO_4$, 50 C_{18}	AOAC 2007.01
	15	8	4000 PSA, 1200 $MgSO_4$, 400 C_{18}	
	2	1	25 PSA, 150 $MgSO_4$, 25 C_{18}	prEN 15662
	15	6	150 PSA, 900 $MgSO_4$, 150 C_{18}	
**Pigmented fruits and vegetables**	2	1	50 PSA, 150 $MgSO_4$, 50 GCB	AOAC 2007.01
	15	8	400 PSA, 1200 $MgSO_4$, 400 GCB	

	2	1	25 PSA, 150 $MgSO_4$, 2.5 GCB	prEN 15662
	15	6	150 PSA, 900 $MgSO_4$, 15 GCB	
**Highly pigmented fruits and vegetables**	2	1	25 PSA, 150 $MgSO_4$, 7.5 GCB	prEN 15662
	15	6	150 PSA, 900 $MgSO_4$, 45 GCB	
**Fruits and vegetables with pigments and fats**	2	1	50 PSA, 150 $MgSO_4$, 50 GCB, 50 C_{18}	AOAC 2007.01
	15	8	400 PSA, 1200 $MgSO_4$, 400 GCB, 400 C_{18}	

Note: dSPE – dispersive solid-phase extraction; PSA - primary secondary amine; GCB - graphitized black carbon; C_{18} - octadecyl modified silica, end-capped; $MgSO_4$ - magnesium sulfate

References

1. EN 15662:2008, Foods of plant origin-determination of pesticide residues using GC-MS and/or LC-MS/MS following acetonitrile, extraction/partitioning and clean-up by dispersive SPE - QuEChERS-method. Available online: http://www.chromnet.net/Taiwan/QuEChERS_Dispersive_SPE/QuEChERS_%E6%AD%90%E7%9B%9F%E6%96%B9%E6%B3%95_EN156622008_E.pdf (accessed on 20 December 2018).
2. Association of Official Analytical Chemists (AOAC) International, AOAC Official Method 2007.01, Pesticide Residues in Foods by Acetonitrile Extraction and Partitioning with Magnesium Sulfate, 2007. Available online: http://www.thenfl.com/pdf/AOAC%202007.1.pdf (accessed on 20 December 2018).
3. Madeja,K.; Kalenikb, T.K.; Piekoszewski, W. Sample preparation and determination of pesticides in fat-containing foods. *Food Chemistry* 2018, 269, 527-541.

Table S2. Fruits and vegetables chosen for the study according to the SANTE guideline [1,2]

Commodity group	Commodity category	Chosen samples	Parameters relevant for the selection of suitable sorbents	Parts of the products for analysis
High water content	Pome fruit	Apple	more than 80 % of water content, presence of organic acids: malic and citric, sugars, proteins, fiber, vitamins	whole product after removing stems and leafs
		Pear	water content more than 80 %, organic acids, sugars	
	Stone fruit	Nectarine	more than 80 % of water content, presence of carotenoids, anthocyanins and polyphenols	
	Fruiting vegetables/cucubits	Tomato	more than 90 % of water content, current carotenoids, vitamins and sugars	

	Cucumber	high water content over 95 %, chlorophyll present, low content of essential nutrients	
	Red pepper	more than 80 % of water content, carotenoid pigments, current vitamins (especially vitamin C, A, B and E), minerals (potassium, calcium), essential oils, polyphenols and sugar	
Brassica vegetables	Broccoli	water content over 80 %, present isothiocyanates, chlorophyll, minerals (potassium, calcium, iron, phosphorus, manganese, magnesium, sulfur), vitamin A (beta-carotene), B_1, B_2, B_6, C, K, PP, pantothenic and folic acids	only florets
Fresh fungi	Mushroom (champignon)	more than 80 % of water content, current minerals and vitamins (B, D and C)	whole product after removal of soil or growing medium
Root and tuber vegetables	Red beet	water content over 80 %, current carotenoids, organic acids (malic, citric, vinous and oxalic), proteins, sugars and vitamins	whole product after removal of tops (if any) and adhering soil by brushing and/or rinsing with distilled water
	Carrot	more than 80 % of water content, present carotenoids, vitamins C, K and B (folic acid, B_1, B_2, B_3, B_5, B_6) and minerals - magnesium, phosphorus, potassium, sodium, copper and manganese	
	Potato	more than 80 % of water content, present carbohydrates (starch), fiber, vitamins (B_1, PP, B_5, B_6, C), minerals (potassium, magnesium,	

		phosphorus, iron, copper, manganese and zinc)
	White radish	high water content, vitamins (C and B), minerals (potassium, sodium, calcium, phosphorus, magnesium, iron, zinc, molybdenum), proteins, carbohydrates, fiber, folic acid and essential oils

References

1. Guidance document on analytical quality control and method validation procedures for pesticides residues analysis in food and feed. SANTE/11813/2017, Available online: https://ec.europa.eu/food/sites/food/files/plant/docs/pesticides_mrl_guidelines_wrkdoc_2017-11813.pdf (accessed on 20 December 2018).

2. United States Department of Agriculture, Agricultural Research Service, USDA Food Composition Databases, https://ndb.nal.usda.gov/ndb/ (accessed on 20 December 2018).
